# The Influence of Polyphenols on Starch Structure Across Molecular, Chemical, and Microstructural Levels

**DOI:** 10.3390/molecules31152700

**Published:** 2026-08-03

**Authors:** Dominika Kwaśny, Barbara Borczak, Paweł Sroka, Katarzyna Walkowiak

**Affiliations:** 1 Department of Human Nutrition and Dietetics, Faculty of Food Technology, University of Agriculture in Krakow, Aleja Mickiewicza 21, 31-120 Krakow, Poland; 2 Department of Fermentation Technology and Microbiology, Faculty of Food Technology, University of Agriculture in Krakow, Aleja Mickiewicza 21, 31-120 Krakow, Poland; 3 Department of Physics and Biophysics, Faculty of Food Science and Nutrition, Poznan University of Life Sciences, Wojska Polskiego 38/42, 60-637 Poznan, Poland

**Keywords:** starch, polyphenol, apparent amylose content, epigallocatechin gallate, SEM, FTIR, digestion

## Abstract

Despite the well-documented effects of polyphenols on the nutritional properties of starch, information regarding the structure of starch–polyphenol complexes remains limited. Therefore, this study aimed to investigate the molecular, chemical, and microstructural properties of starch-polyphenol complexes prepared from wheat, rice, potato, and maize starches. Apparent amylose content was determined spectrophotometrically, molecular interactions were analyzed using Fourier Transform Infrared (FTIR) spectroscopy, and morphological changes were evaluated by scanning electron microscopy (SEM). Significant differences in apparent amylose content were observed following the incorporation of selected polyphenols, especially epigallocatechin gallate. FTIR analysis suggested that polyphenols may alter the molecular organization of starch matrices through non-covalent interactions, predominantly hydrogen bonding. Epigallocatechin gallate and quercetin exerted more noticeable effects than the other polyphenols studied. Furthermore, SEM observations performed exclusively on starch systems containing epigallocatechin gallate revealed noticeable morphological alterations in native starch granules following in vitro digestion compared with undigested controls. These findings provide new insights into the formation of starch-polyphenol complexes and support their potential application in the development of functional foods with improved nutritional properties.

## 1. Introduction

Non-communicable diseases, particularly type 2 diabetes mellitus, represent one of the most significant global public health challenges. Type 2 diabetes accounts for more than 90% of all diabetes cases and is closely associated with obesity and other diet-related chronic diseases. Among the dietary factors contributing to the development and progression of diabetes, the consumption of high-glycemic-index foods plays a crucial role, as it leads to rapid increases in postprandial blood glucose levels [1,2,3,4].

Starch is the primary source of carbohydrates in the human diet and typically provides a substantial proportion of daily energy intake. However, many commonly consumed starch-rich foods, such as cereal-based products, have a high glycemic index. Since the physiological response to starch depends not only on its quantity but also on the rate and extent of its digestion, strategies aimed at reducing starch digestibility have attracted considerable scientific interest. Modifying starch digestion may improve glycemic control and, consequently, prevent and manage metabolic disorders [5,6,7]. 

Polyphenols are naturally occurring bioactive compounds widely distributed in plant-derived foods and are recognized for their numerous health-promoting properties. In addition to their antioxidant activity, polyphenols have been shown to reduce starch digestibility through interactions with digestive enzymes, particularly α-amylase, as well as through direct interactions with starch molecules. These interactions are mainly mediated by hydrogen bonding and hydrophobic forces, resulting in the formation of starch–polyphenol complexes that are less susceptible to enzymatic hydrolysis [8,9,10].

The formation of starch–polyphenol complexes has emerged as a promising approach for developing functional foods with improved nutritional properties. Previous studies [11,12,13] have demonstrated that such complexes may decrease starch digestibility, increase resistant starch content, lower the estimated glycemic index, and enhance antioxidant activity. They can also affect starch cytotoxicity. Consequently, starch–polyphenol complexes are increasingly considered as potential ingredients for food formulation to support the prevention and management of type 2 diabetes and other metabolic disorders. In the present study, starch–polyphenol complexes were characterized at the molecular, chemical, and microstructural levels by evaluating apparent amylose content, starch morphology, and the intermolecular interactions occurring within the complexes.

The amylose content depends on the plant source and influences the structural and morphological characteristics of starch, including molecular weight, granule shape, and the balance between crystalline and amorphous regions [14]. The starch composition of major food crops such as wheat, rice, potato, and maize typically comprises approximately 20–30% amylose and 70–80% amylopectin [15,16]. An increase in amylose content relative to amylopectin has been shown to increase resistant starch content and reduce the glycemic index of starch [11,15,17]. 

Starch interacts with polyphenols through non-covalent interactions, including hydrogen bonding, hydrophobic interactions, and electrostatic and ionic interactions. These interactions lead to the formation of two types of complexes: inclusion, with the V-type inclusion complex being the most commonly observed, and non-inclusion complexes. Inclusion complexes are generally characterized by stronger binding interactions than non-inclusion complexes [18,19]. Molecular interactions and accompanying changes in the molecular organization of starch–polyphenol complexes can be effectively characterized by Fourier Transform Infrared (FTIR) spectroscopy. These interactions may alter the ordered structure, crystallinity, and digestibility of starch [19,20].

The interaction between starch and polyphenols may result in modifications in starch morphology and granule microstructure [19]. Scanning electron microscopy (SEM) is commonly applied to characterize these structural changes, including surface erosion, pore formation, and granule deformation [21,22]. Such morphological alterations may indicate the formation of starch–polyphenol complexes and may be linked to changes in the physicochemical characteristics and digestibility of starch systems [19,23].

Therefore, the aims of the present study were to: (1) determine the apparent amylose content of starch–polyphenol complexes, (2) investigate the molecular interactions occurring within the complexes, and (3) evaluate the morphological changes in starch following in vitro digestion in the presence of the polyphenol. 

To visualize starch granule morphology and digestion-induced surface modifications, SEM was performed on native starch granules, as gelatinized samples were unsuitable for meaningful morphological evaluation.

## 2. Results and Discussion

### 2.1. Apparent Amylose Content of Starch–Phenolic Complexes

In wheat starch, the highest apparent amylose content was observed in the control sample (31.20%, *p* < 0.05; Table 1). The amylose content of conventional wheat starch generally ranges from 25% to 28% [24,25], although values between 0% and 38% have been reported depending on wheat species and cultivar [24]. The value obtained in the present study was therefore consistent with previously reported data.

It has been established that polyphenols do not modify the chemical composition of amylose, but they can impact its availability and structure. Due to the linear structure of amylose, polyphenols exhibit a greater affinity for this fraction than for branched amylopectin. The highest apparent amylose content observed in the control sample may be attributable to the capacity of amylose to form non-covalent inclusion complexes with a diverse array of small molecules, including polyphenols. Compared with amylopectin, amylose has a longer chain length, which consequently results in a greater capacity to form complexes with guest molecules. The capacity of amylose to form complexes with polyphenols may limit its analytical accessibility and, consequently, result in an apparent decline in its quantity [14,26,27].

For rice starch, the sample containing epigallocatechin gallate exhibited the highest mean apparent amylose content (26.95%, *p* < 0.05, Table 2), although the control sample belonged to an overlapping statistical group (21.99%, *p* < 0.05, Table 2). According to the literature, the amylose content of rice starch is highly dependent on the cultivar and may vary from 0% to 37% [28,29]. In general, conventional rice starch contains between 13% and 27% amylose [30]. Therefore, the values obtained in the present study were within the range previously reported for rice starch.

The higher apparent amylose content observed in the presence of epigallocatechin gallate, in comparison to the other polyphenols studied (hesperidin, naringenin, *trans*-ferulic acid, *p*-coumaric acid, quercetin, and kaempferol), may be due to its specific chemical structure, which is rich in hydroxyl groups that promote strong interactions with starch (both amylose and amylopectin). It has been determined that an increase in hydrogen bonds can hinder intermolecular interactions among starch chains, thereby altering starch structure. Moreover, the polyphenol exhibits one of the largest molecular sizes among the investigated polyphenolic compounds. Additionally, epigallocatechin gallate may destabilize the ordered structure of amylopectin and reduce starch crystallinity, thereby increasing the availability of amylose. The exact mechanism underlying the observed differences in apparent amylose content remains unclear and cannot be conclusively determined from the data obtained in the present study. The decrease in apparent amylose content observed in some starch–polyphenol systems may be related to the formation of starch–polyphenol interactions that affect the analytical accessibility of amylose. Conversely, the higher apparent amylose content observed in rice starch containing epigallocatechin gallate may reflect structural rearrangements within the starch matrix that increase the accessibility of amylose during analysis. However, these explanations remain hypothetical and require further investigation using complementary structural techniques [14,19,31].

It has been demonstrated that the starch–polyphenol complex containing epigallocatechin gallate exhibits not only a higher apparent amylose content but also enhanced properties. As previously outlined in our preceding articles [11,12,13], the chemical structure of epigallocatechin gallate renders it the most potent inhibitor of starch digestibility (including rice starch) among the polyphenols studied. In addition, it was identified as one of the polyphenols that, upon complexation, resulted in the highest antioxidant activity in starch (including rice starch).

Among the investigated samples, the *p*-coumaric acid-treated starch showed the highest mean apparent amylose content (22.66%, *p* < 0.05, Table 3), whereas the naringenin-treated sample showed the lowest mean value (15.39%, *p* < 0.05, Table 3). Most of the remaining samples belonged to overlapping statistical groups and therefore were not significantly different from either extreme (Table 3). The remaining starch–polyphenol complexes showed intermediate apparent amylose contents and belonged to overlapping statistical groups. According to the literature, the amylose content of potato starch typically ranges from 11.9% to 20.1% [32,33]. Thus, most of the values obtained in the present study fell within or near the previously reported range for potato starch.

The differences observed between *p*-coumaric acid and naringenin in their interactions with potato starch can be explained by their structural characteristics. *p*-coumaric acid exhibits relatively weak interactions with starch due to its small molecular size and only one hydroxyl group in its molecular structure. In contrast, naringenin can form stronger interactions with starch through multiple hydrogen bonds, resulting in more stable complexes. Such interactions may induce structural rearrangements within starch granules, thereby influencing amylose accessibility [14,19,31].

For maize starch, the sample containing epigallocatechin gallate exhibited the highest mean apparent amylose content (30.27%; *p* < 0.05, Table 4), whereas the samples treated with *trans*-ferulic acid and quercetin showed the lowest mean apparent amylose contents (19.10% and 22.19%, respectively; *p* < 0.05, Table 4). The remaining samples belonged to overlapping statistical groups (Table 4). According to the literature, the amylose content of maize starch varies considerably by genotype, ranging from 0% to more than 33%. Conventional maize starch typically contains 25–28% amylose [25,30,34]. Therefore, the apparent amylose contents determined in the present study were generally consistent with the ranges reported in previous studies.

The higher apparent amylose content observed in maize starch in the presence of epigallocatechin gallate, compared to *trans*-ferulic acid and quercetin, may be attributed to its stronger interactions with starch. The interaction strength between starch and phenolic compounds increases with the number of hydroxyl groups and molecular size, making epigallocatechin gallate particularly effective. Moreover, epigallocatechin gallate can disrupt the ordered structure of starch and reduce its crystallinity, thereby affecting amylopectin organization and increasing amylose accessibility. In contrast to epigallocatechin gallate, *trans*-ferulic acid contains only one hydroxyl group in its molecular structure, which limits its ability to form hydrogen bonds and results in weaker and less stable interactions with starch [14,19,31]. Conversely, the planar molecular structure of quercetin may promote the formation of strong, highly ordered, and compact complexes, thereby limiting access to amylose [35,36].

It is worth noting that the direction of the observed changes in apparent amylose content varied among the starches investigated. These contrasting responses indicate that the effect of polyphenols on apparent amylose content is starch-dependent. The observed differences may reflect variations in the structural organization of starches from different botanical sources rather than a common interaction mechanism. Further studies are required to elucidate the molecular basis of these starch-specific responses.

One limitation of the present study is the absence of a solvent-only control group. Consequently, the observed differences in apparent amylose content cannot be unequivocally attributed to structural modifications of starch. The changes may instead reflect altered analytical accessibility of amylose resulting from starch–polyphenol complex formation, which could partially mask amylose during the analytical procedure. Therefore, the results should be interpreted as changes in apparent amylose content rather than direct evidence of changes in the absolute amylose concentration.

### 2.2. Molecular and Chemical Interactions in Starch–Phenolic Complexes

The FTIR spectra obtained for the investigated starch–polyphenol systems after in vitro digestion suggested that the incorporation of phenolic compounds influenced the supramolecular organization of starch matrices. The observed spectral modifications in the post-digestion residues were primarily associated with changes in hydrogen-bond interactions, alterations in water organization, and partial reorganization of the amorphous and crystalline regions of the polysaccharide structure. The magnitude of these effects depended strongly on both the botanical origin of the starch and the chemical structure of the incorporated phenolic compounds.

A limitation of the present study is that the FTIR spectra were acquired after in vitro digestion. Consequently, the spectra reflect the molecular characteristics of the residual material remaining after enzymatic hydrolysis rather than those of the intact starch–polyphenol complexes. Therefore, the FTIR findings should be interpreted within the context of the post-digestion residues and should not be regarded as a direct characterization of the native starch–polyphenol systems.

In all investigated systems, noticeable spectral changes were observed within the hydroxyl stretching region (3600–3000 cm^−1^) and within the characteristic polysaccharide fingerprint region (1200–900 cm^−1^). These observations indicate that the effects of polyphenol incorporation on intermolecular interactions and molecular ordering remained detectable in the residual starch material after in vitro digestion, rather than indicating permanent modification of starch chains.

The broadening and increased intensity of hydroxyl-related bands suggest intensified hydrogen-bond formation between starch hydroxyl groups and phenolic compounds. Polyphenols containing multiple hydroxyl substituents, such as epigallocatechin gallate and quercetin, were associated with more evident interactions due to their high ability to act as both hydrogen-bond donors and acceptors. Simultaneously, the increase in the intensity of the water-associated band near 1650 cm^−1^ indicates enhanced stabilization of bound water molecules within the starch matrix.

Changes observed within the fingerprint region suggest partial disruption of molecular ordering and local reorganization of amylose and amylopectin chains. However, the absence of new absorption bands characteristic of covalent bonds is consistent with the modification process remaining predominantly physical.

The FTIR spectra were interpreted qualitatively based on changes in band positions, intensities, and shapes. Therefore, the FTIR results should be interpreted as qualitative evidence of starch–polyphenol interactions rather than direct proof of changes in starch molecular organization or crystallinity. The present study did not include quantitative evaluation of the short-range molecular order using absorbance ratios such as 1047/1022 cm^−1^ and 1022/995 cm^−1^, nor spectral deconvolution analysis. Future studies incorporating these quantitative parameters, together with complementary structural techniques such as XRD or DSC, would provide a more comprehensive characterization of starch–polyphenol interactions.

#### 2.2.1. FTIR Analysis of Wheat Starch

**Figure 1:**
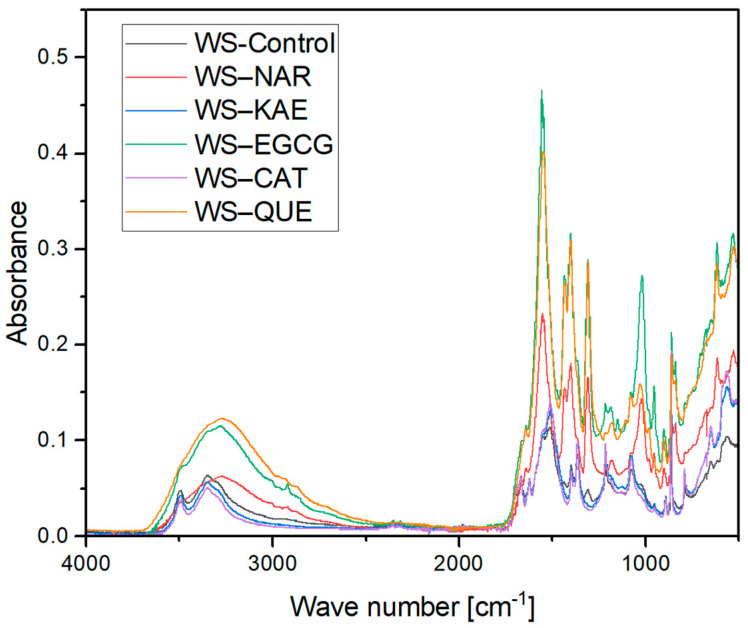
Fourier Transform Infrared (FTIR) spectra analysis of wheat starch and polyphenol-modified starch. WS-Control: wheat starch; WS-NAR: naringenin-wheat starch complex; WS-KAE: kaempferol-wheat starch complex; WS-EGCG: epigallocatechin gallate-wheat starch complex; WS-CAT: (+)-catechin-wheat starch complex; WS-QUE: quercetin-wheat starch complex.

The FTIR spectra of wheat starch (WS-Control) and polyphenol-modified samples after digestion are presented in Figure 1. A broad absorption band within the range of 3600–3000 cm^−1^ was observed for all samples and was assigned to the stretching vibrations of hydroxyl groups and bound water molecules. Polyphenol addition resulted in band broadening and increased intensity, particularly for samples containing epigallocatechin gallate (WS-EGCG) and quercetin (WS-QUE), suggesting the presence of hydrogen-bond interactions that persisted in vitro digestion [19,37].

In the region of 3000–2800 cm^−1^ corresponding to ν(C-H) vibrations, no significant band shifts were detected. However, slight increases in signal intensity were observed for WS-EGCG and WS-QUE, suggesting changes in the local chemical environment without covalent modification of the polysaccharide structure.

The band located at approximately 1650–1640 cm^−1^, assigned to bending vibrations of bound water molecules, exhibited increased intensity after polyphenol incorporation. The strongest effect was observed in the WS-EGCG and WS-QUE samples, suggesting altered water organization in the residual starch material [38,39].

The relatively greater changes were observed in the 1200–900 cm^−1^ region corresponding to C-O, C-O-C, and C-C vibrations characteristic of the polysaccharide structure. Polyphenol-modified samples exhibited band broadening, changes in intensity, and slight shifts in absorption maxima. The strongest spectral disturbances were detected in WS-EGCG and WS-QUE, suggesting residual non-covalent interactions that may have contributed to the structural organization of the residual starch material after digestion [19,40].

#### 2.2.2. FTIR Analysis of Rice Starch

**Figure 2:**
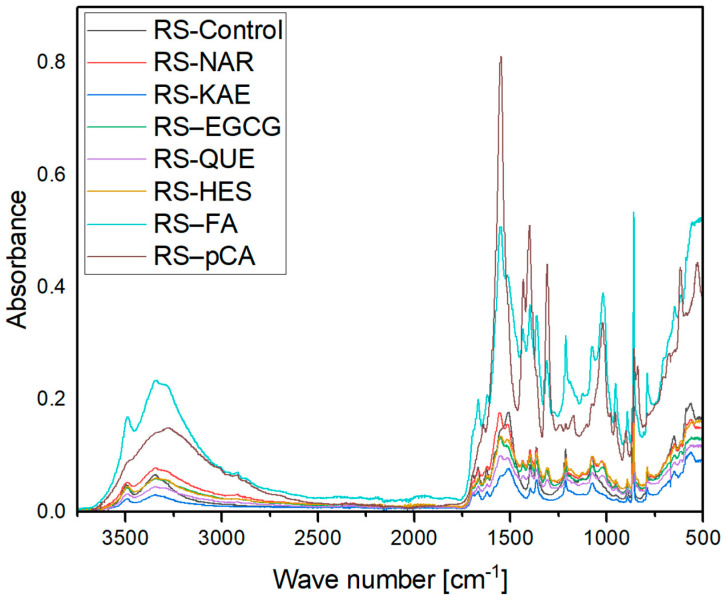
FTIR spectra analysis of rice starch and polyphenol-modified starch. RS-Control: rice starch; RS-NAR: naringenin-rice starch complex; RS-KAE: kaempferol-rice starch complex; RS-EGCG: epigallocatechin gallate-rice starch complex; RS-QUE: quercetin-rice starch complex; RS-HES: hesperidin-rice starch complex; RS-FA: *trans*-ferulic acid-rice starch complex; RS-pCA: *p*-coumaric acid-rice starch complex.

The FTIR spectra of rice starch and polyphenol-containing systems are presented in Figure 2. All samples exhibited a characteristic broad band in the 3600–3000 cm^−1^ region associated with hydroxyl group vibrations. Polyphenol incorporation increased the intensity of this band, especially in samples containing epigallocatechin gallate, quercetin, and *trans*-ferulic acid, indicating intensified hydrogen-bond interactions in the post-digestion residues.

An increase in the intensity of the band at approximately 1650 cm^−1^ was observed in samples containing epigallocatechin gallate (RS-EGCG), *trans*-ferulic acid (RS-FA), and *p*-coumaric acid (RS-*p*CA), suggesting enhanced water-binding capacity and reorganization of the amorphous structure [39].

Additional bands in the 1600–1500 cm^−1^ region corresponding to aromatic ring vibrations were consistent with the presence of phenolic compounds in the starch matrix into the starch matrix. More prominent signals were observed for systems containing quercetin and epigallocatechin gallate.

Changes in the 1200–900 cm^−1^ region indicate partial structural reorganization of the residual rice starch following digestion. The most noticeable spectral disturbances were observed for samples containing epigallocatechin gallate (RS-EGCG), quercetin (RS-QUE), and *trans*-ferulic acid (RS-FA), suggesting that interactions between polyhydroxylated polyphenols and starch remained detectable after digestion.

#### 2.2.3. FTIR Analysis of Potato Starch

**Figure 3:**
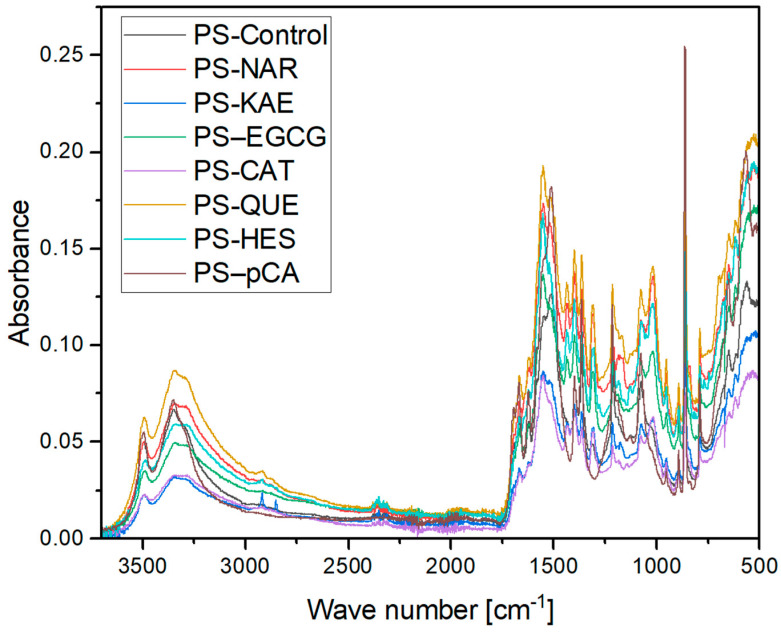
FTIR spectra analysis of potato starch and polyphenol-modified starch. PS-Control: potato starch; PS-NAR: naringenin-potato starch complex; PS-KAE: kaempferol-potato starch complex; PS-EGCG: epigallocatechin gallate-potato starch complex; PS-CAT: (+)-catechin-potato starch complex; PS-QUE: quercetin-potato starch complex; PS-HES: hesperidin-potato starch complex; PS-pCA: *p*-coumaric acid-potato starch complex.

The FTIR spectra of potato starch and polyphenol-modified samples are shown in Figure 3. Significant broadening and increased intensity of the ν(-OH) band within 3600–3000 cm^−1^ were observed, particularly for samples containing epigallocatechin gallate (PS-EGCG), quercetin (PS-QUE), and hesperidin (PS-HES). These changes indicate that intensified hydrogen-bond interactions between polyphenols and the starch matrix remained detectable after in vitro digestion [19,37].

The band near 1650 cm^−1^ exhibited markedly increased intensity after incorporation of epigallocatechin gallate and quercetin, suggesting enhanced water-binding capacity. Additional signals observed within the 1600–1500 cm^−1^ region suggested that phenolic compounds remained associated with the residual starch material after in vitro digestion. 

The most noticeable changes were detected in the 1200–900 cm^−1^ region. Alterations in band shape and intensity suggest supramolecular reorganization of potato starch and partial disruption of crystalline order. These effects were particularly evident for samples containing epigallocatechin gallate, catechin, and quercetin.

#### 2.2.4. FTIR Analysis of Maize Starch

**Figure 4:**
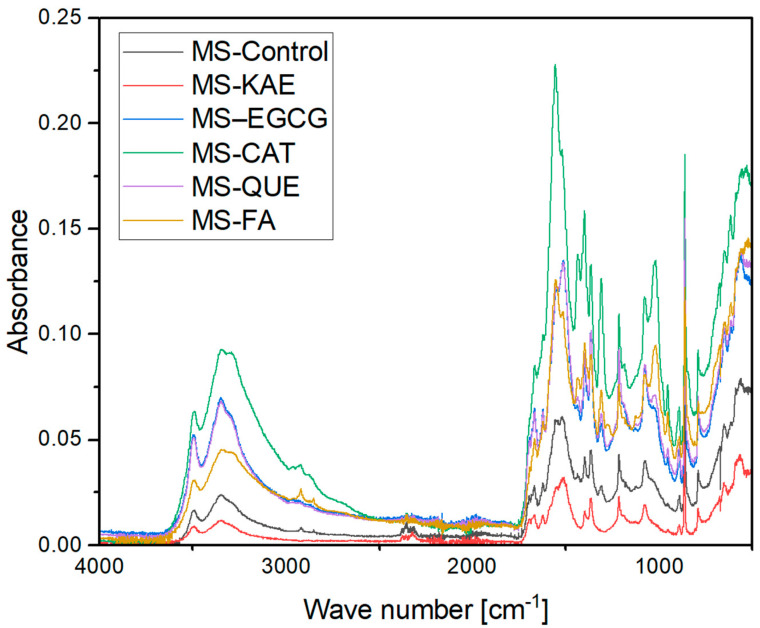
FTIR spectra analysis of maize starch and polyphenol-modified starch. MS-Control: maize starch; MS-KAE: kaempferol-maize starch complex; MS-EGCG: epigallocatechin gallate-maize starch complex; MS-CAT: (+)-catechin-maize starch complex; MS-QUE: quercetin-maize starch complex; MS-FA: *trans*-ferulic acid-maize starch complex.

The FTIR spectra of maize starch and polyphenol-modified systems are presented in Figure 4. The control sample exhibited a broad band characteristic of hydroxyl group stretching vibrations. Polyphenol addition caused increased intensity and broadening of this band, especially for samples containing epigallocatechin gallate (MS-EGCG) and (+)-catechin (MS-CAT), indicating the presence of an extensive hydrogen-bonding network in the post-digestion residues [37,40].

The band around 1645 cm^−1^ showed increased intensity in samples containing epigallocatechin gallate, catechin, and quercetin, suggesting enhanced water-binding capacity and partial reorganization of the amorphous phase.

Within the 1600–1500 cm^−1^ region, characteristic bands corresponding to aromatic ring vibrations appeared, particularly in samples containing quercetin (MS-QUE) and *trans*-ferulic acid (MS-FA). The absence of substantial changes in ν(C-H) vibrations indicates that no permanent chemical modifications occurred within the polysaccharide backbone.

The 1200–900 cm^−1^ region exhibited significant spectral alterations, including changes in band intensity and broadening. These findings suggest structural rearrangements remaining after digestion that are consistent with non-covalent starch–polyphenol interactions.

The FTIR results suggest that interactions between phenolic compounds and starch remained detectable after in vitro digestion and were primarily associated with non-covalent mechanisms, including hydrogen bonding and hydrophobic interactions. The effects were especially evident for compounds rich in hydroxyl groups, such as epigallocatechin gallate and quercetin, which is consistent with previous reports regarding starch–polyphenol complex formation [19,37,39].

Changes in the intensity of the band around 1650 cm^−1^ suggest altered organization of bound water in the residual starch material after in vitro digestion, which may be associated with the presence of polyphenols.

Increased intensity of this signal suggests enhanced hydrophilicity and partial reorganization of the amorphous starch phase.

Potato and maize starches were the most susceptible to structural modification, likely due to greater accessibility of the amorphous phase and lower crystalline order. In contrast, rice starch exhibited a more stable spectral profile, suggesting less pronounced structural changes remained after digestion.

### 2.3. Morphological Structure of Starch–Phenolic Complexes

A limitation of the present study is that SEM observations were performed using native starches rather than gelatinized systems. Although this approach enabled visualization of granule morphology and digestion-induced surface alterations, it does not fully reflect the form in which starch is typically consumed. Consequently, SEM results should be interpreted primarily as evidence of starch–polyphenol interactions and morphological changes occurring at the granule level rather than as a direct representation of physiological digestion. Therefore, these observations should not be directly extrapolated to gelatinized or cooked starch systems, as the structural organization and enzymatic hydrolysis behavior of gelatinized starch differ substantially from those of native starch granules.

Another limitation of the present study is that SEM was used exclusively for qualitative visualization. Therefore, quantitative morphometric analysis of starch granule size with statistical evaluation (mean ± SD, *n*) was not performed.

#### 2.3.1. SEM Images of Wheat Starch

Starch is deposited in amyloplasts as discrete granules that vary in size and morphology during grain development. In wheat, granules show a trimodal size distribution, comprising large A-, intermediate B-, and small C-type fractions. A-granules (10–40 µm; typically 23–28 µm) are lenticular and disc-shaped, whereas B- and C-granules are smaller (≈9–11 µm and 2–3 µm, respectively) and exhibit more spherical, oval, or irregular forms. In some classifications, B- and C-granules are grouped as small granules [24,28].

**Figure 5:**
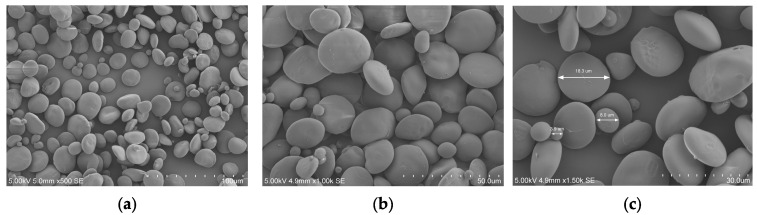
Scanning electron microscopy (SEM) images of undigested wheat starch at magnification 500× (**a**), 1000× (**b**), and 1500× (**c**).

In the present study (Figure 5), both smaller and larger starch granules could be distinguished. The larger granules were morphologically consistent with A-type granules, whereas the smaller ones resembled B- and C-type granules. Representative dimensions (e.g., approximately 8.0 and 3.9 μm, Figure 5c) are provided solely to illustrate the observed morphology and should not be interpreted as quantitative measurements. Furthermore, the larger granules exhibited a lenticular, disc-shaped morphology, while the smaller granules appeared more rounded and spherical.

**Figure 6:**
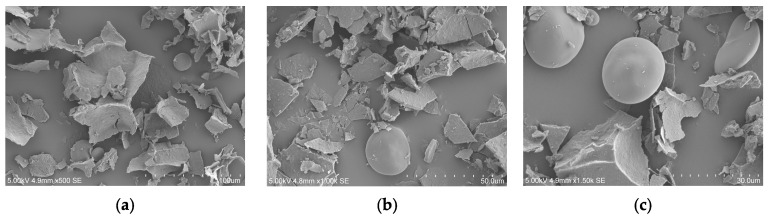
SEM images of digested wheat starch with the polyphenol at magnification 500× (**a**), 1000× (**b**), and 1500× (**c**).

**Figure 7:**
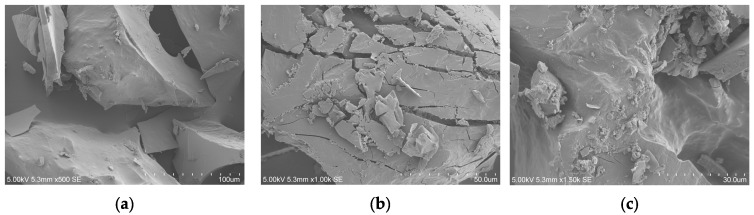
Representative SEM image of enzymatically digested starch without polyphenol, presented as a reference illustrating the general morphology of the digested control.at magnification 500× (**a**), 1000× (**b**), and 1500× (**c**).

**Figure 8:**
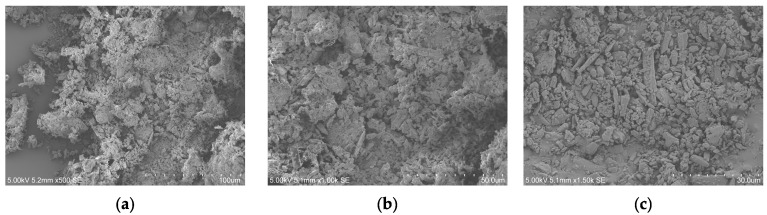
SEM images of epigallocatechin gallate at magnification 500× (**a**), 1000× (**b**), and 1500× (**c**).

Among the polyphenols examined in previous studies [12], epigallocatechin gallate showed the strongest inhibitory effect on starch digestion. Distinct morphological features of the starch granules can also be observed in the corresponding SEM images presented in Figure 6. Notably, certain starch granules remained intact, showing no signs of partial digestion or surface degradation and retaining a morphology comparable to that of undigested granules (Figure 5). In contrast, no recognizable starch granules were observed in the digested control sample without polyphenol addition (Figure 7), indicating that the starch was almost completely hydrolyzed during digestion. The remaining structures visible in the images are most likely residues of the digestion mixture. However, these structures did not resemble the morphology of epigallocatechin gallate (Figure 8), most likely due to dissolution of the compound in the digestion mixture, followed by its removal during decantation in the SEM sample preparation process.

#### 2.3.2. SEM Images of Rice Starch

In mature grains, rice starch granules are typically small and cuboidal, generally smaller than those of wheat or maize. They commonly occur as aggregates of tightly associated granules within the endosperm and are therefore often described as compound starch granules [28].

**Figure 9:**
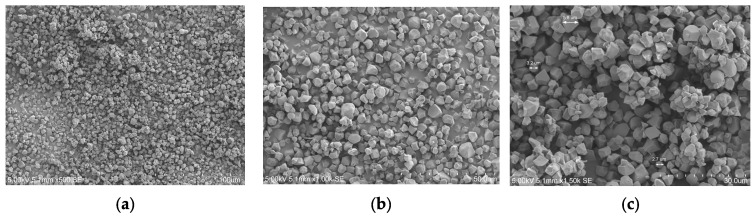
SEM images of undigested rice starch at magnification 500× (**a**), 1000× (**b**), and 1500× (**c**).

The findings of the present study (Figure 9) are consistent with previous reports in the literature. Rice starch granules were considerably smaller compared with those of wheat, potato, and maize starches and were observed as compact aggregates of closely associated granules with predominantly cuboidal shapes. Representative dimensions of selected granules (approximately 5.8, 3.2, and 2.7 µm; Figure 9c) are provided solely to illustrate the observed morphology and should not be interpreted as the results of a comprehensive quantitative size analysis.

**Figure 10:**
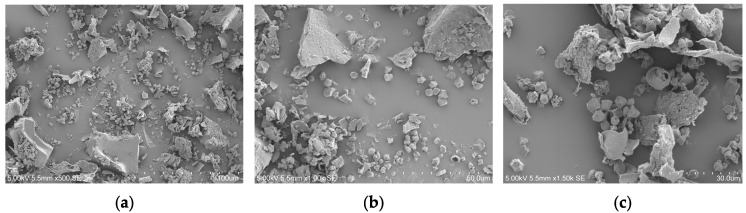
SEM images of digested rice starch with the polyphenol at magnification 500× (**a**), 1000× (**b**), and 1500× (**c**).

Similar to wheat starch, SEM images of digested rice starch in the presence of the polyphenol revealed intact starch granules (Figure 10). In addition, partially digested granules with disrupted surface morphology were also observed, sometimes revealing the layered cross-section of the starch granule or a digested interior with only the outer layers remaining intact. In contrast, no identifiable starch granules were detected in the digested control sample without polyphenol addition (Figure 7), indicating extensive starch hydrolysis during digestion.

According to the literature, the concentration of the enzyme used to digest starch can affect the morphological changes observed in starch granules. SEM images obtained at various magnifications revealed noticeable alterations in granule morphology after enzymatic treatment compared to native rice starch. At higher α-amylase activity, the granule surfaces exhibited numerous small pores and erosion sites. In contrast, lower enzyme concentrations resulted mainly in slight surface undulations without complete penetration into the granule structure [41]. 

Another SEM analysis revealed numerous small pores on the surface of starch granules subjected to amylase digestion. In some granules, the pores penetrated the inner regions, producing a terraced or layered appearance indicative of the internal layered structure of starch granules [42].

#### 2.3.3. SEM Images of Potato Starch

The predominant granule size fraction in the potato starch was found to range between 10 and 12 μm. Potato starch exhibited a broad range of particle morphologies, including spherical, oval, and irregular polygonal shapes. These structural characteristics were generally maintained after tablet compaction; however, some particles exhibited fragmentation and surface cracks [43,44].

**Figure 11:**
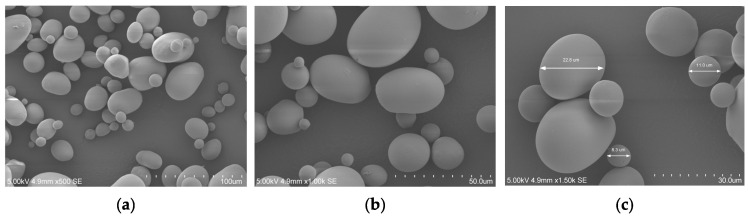
SEM images of undigested potato starch at magnification 500× (**a**), 1000× (**b**), and 1500× (**c**).

In the present study, potato starch granules displayed predominantly spherical and oval shapes (Figure 11). Considerable variation in granule size was also observed. Representative dimensions of selected granules (approximately 22.8, 11.0, and 8.3 μm; Figure 11c) are provided solely to illustrate the observed morphology and should not be interpreted as the results of a comprehensive quantitative analysis of granule size distribution.

**Figure 12:**
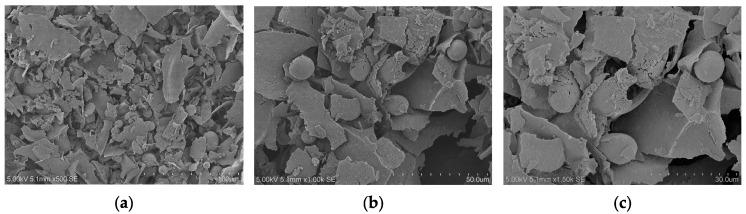
SEM images of digested potato starch with the polyphenol at magnification 500× (**a**), 1000× (**b**), and 1500× (**c**).

Similar to wheat and rice starch digested in the presence of epigallocatechin gallate, undigested potato starch granules were also observed (Figure 12). Some granules appeared slightly overdigested or exhibited distinct surface irregularities. By comparison, the control sample digested in the absence of polyphenols (Figure 7) contained no discernible starch granules.

Compared with wheat, rice, and maize starch, potato starch appears to be more resistant to degradation during enzymatic digestion [45]. Detailed SEM analysis of native and hydrolyzed potato starch demonstrated that the outer shell of the granules exhibits greater flexibility and resistance to mechanical disruption. At the same time, the internal blocklets expand during heat-moisture treatment and may protrude through the outer layer [46].

#### 2.3.4. SEM Images of Maize Starch

Maize starch was predominantly composed of granules measuring 8–10 μm in diameter. As observed for potato starch, maize starch displayed considerable morphological diversity, with granules exhibiting spherical, oval, and irregular polygonal forms. Following tablet compaction, these structural characteristics were largely preserved, although some granules exhibited fragmentation and surface fissures [43,44].

**Figure 13:**
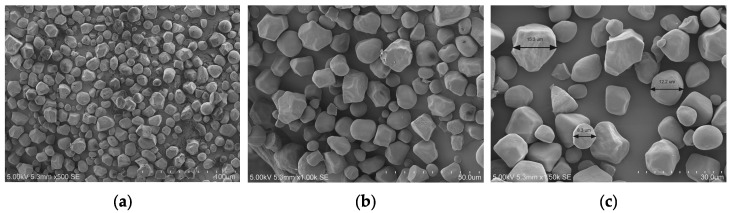
SEM images of undigested maize starch at magnification 500× (**a**), 1000× (**b**), and 1500× (**c**).

Maize starch granules examined in the present study exhibited diverse morphologies, including spherical, oval, and polygonal shapes (Figure 13). Considerable variation in granule size was also observed. Representative dimensions of selected granules (approximately 8.3, 12.2, and 15.3 μm; Figure 13a) are provided solely to illustrate the observed morphology and should not be interpreted as quantitative morphometric measurements or as the results of a comprehensive quantitative analysis of granule size distribution.

**Figure 14:**
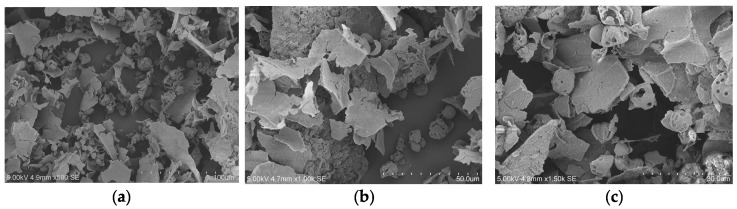
SEM images of digested maize starch with the polyphenol at magnification 500× (**a**), 1000× (**b**), and 1500× (**c**).

According to the literature, enzymatic hydrolysis of starch induced noticeable surface alterations, characterized by the formation of pores on most starch granules while largely preserving their overall shape [47]. Similarly, in the present study, digestion of maize starch in the presence of the polyphenol resulted in the formation of numerous pores on the granule surface and degradation of the internal layers (Figure 14). This observation contrasts with the digested control sample (Figure 7), in which no intact starch granules could be identified.

## 3. Materials and Methods

### 3.1. Sample Preparation (Apparent Amylose Content and FTIR Analysis)

#### 3.1.1. Formation of Starch–Phenolic Compound Conjugates

Aqueous starch dispersions (5% *w*/*v*) were prepared using wheat, rice, potato, and maize starches (Sigma-Aldrich S5127; Sigma-Aldrich S7260, St. Louis, MO, USA; Chempur, Piekary Śląskie, Poland; Biomus, Lublin, Poland, respectively). In addition to starch, eight polyphenolic compounds representing four chemical classes were selected: (*+*)-catechin hydrate (Sigma Aldrich 22110, St. Louis, MO, USA) and epigallocatechin gallate (Sigma Aldrich PHR1333, St. Louis, MO, USA) (flavanols), quercetin (Sigma Aldrich Q4951, St. Louis, MO, USA) and kaempferol (Sigma Aldrich K0133, St. Louis, MO, USA) (flavonols), naringenin (Sigma Aldrich N5893, St. Louis, MO, USA) and hesperidin (Sigma Aldrich H5254, St. Louis, MO, USA) (flavanones), as well as *trans*-ferulic acid (Sigma Aldrich 128708, St. Louis, MO, USA) and *p*-coumaric acid (Sigma Aldrich C9008, St. Louis, MO, USA) (phenolic acids). Each polyphenol was incorporated individually into the starch system using two different approaches: either before gel formation (polyphenol addition followed by starch gelatinization at 90 °C and subsequent cooling to 37 °C) or after gel preparation (starch gelatinization at 90 °C, cooling to 37 °C, and then polyphenol addition). The compounds were applied at concentrations of 5, 10, and 20 mg [12].

#### 3.1.2. Selection of Samples for the Present Investigation

The concentrations of individual polyphenols used throughout this study, including those used in the determination of apparent amylose content, FTIR spectroscopy, and SEM analysis, were selected based on our previous study [12]. These concentrations produced the most pronounced effects on starch digestibility and estimated glycemic index and were therefore considered representative for further molecular, chemical, and microstructural characterization. Each analysis was performed using samples prepared from a separate batch of the corresponding starch-polyphenol system (see Table 5).

### 3.2. Measurement of Apparent Amylose Content of Starch–Phenolic Complexes

The apparent amylose content of starch samples was determined using a commercial enzymatic assay kit (Amylose/Amylopectin Assay Kit, Megazyme Ltd., Bray, Ireland; K-AMYL), based on the selective precipitation of amylopectin with concanavalin A (Con A), followed by enzymatic hydrolysis and spectrophotometric quantification of the released glucose using a glucose oxidase–peroxidase (GOPOD) system [48]. The method is based on a modification of the Con A procedure originally described by Yun and Matheson [49] and further developed by Gibson et al. [50]. Starch–polyphenol samples (25 mg) were dispersed in dimethyl sulfoxide (DMSO) (Chempur, Piekary Śląskie, Poland) and heated in a boiling water bath (MEMMERT WNE 14, Schwabach, Germany) to ensure complete solubilization. Lipids were removed by precipitation with 96% (*v*/*v*) ethanol (Stanlab, Lublin, Poland), followed by centrifugation (MPW MED. INSTRUMENTS 351e, Warszawa, Poland) and recovery of the starch pellet. The pellet was redissolved in DMSO, heated, and diluted with Con A buffer to obtain the stock solution. An aliquot of the solution was mixed with Con A and incubated at room temperature for 1 h to selectively precipitate amylopectin. After centrifugation, the supernatant containing amylose was collected, treated with sodium acetate buffer, and enzymatically hydrolyzed using a mixture of pancreatic α-amylase and amyloglucosidase (PAA (40 KU/g)/AMG (17 KU/g)). The released glucose was quantified using a glucose oxidase–peroxidase (GOPOD) assay by measuring absorbance at 510 nm (SPECORD 40, Analytik Jena GmbH, Jena, Germany). Total starch content was determined in parallel by enzymatic hydrolysis of a separate aliquot without Con A treatment, followed by glucose determination under identical conditions. Apparent amylose content was calculated from the absorbance ratio.

Apparent amylose content results were expressed as ranges obtained from at least three independent replicates, with standard deviation calculated relative to the mean. One-way analysis of variance (ANOVA) was used to evaluate the effects of different polyphenolic compounds on the apparent amylose content of various starch types. Statistical differences between groups were identified using Duncan’s multiple range test at a significance level of *p* < 0.05. All statistical computations were carried out using Statistica version 13 software (StatSoft, Inc., Tulsa, OK, USA).

### 3.3. Analysis of Starch–Phenolic Complexes’ Molecular and Chemical Interactions

The samples were subsequently digested in vitro using pancreatic α-amylase and amyloglucosidase (PAA (40 KU/g)/AMG (17 KU/g)) prepared in 50 mM sodium maleate buffer (pH 6.0) containing 2 mM CaCl_2_. Digestion was carried out at 37 °C for 4 h in a thermostatically controlled water bath (MEMMERT WNE 14, Schwabach, Germany) with continuous shaking (170 rpm), enabling enzymatic hydrolysis of starch to *D*-glucose. The samples were freeze-dried. Hydrogen bonding in the analyzed starch samples of different botanical origins enriched with polyphenols was assessed using Fourier-transform infrared (FTIR) spectroscopy. Measurements were performed at the Poznan University of Life Sciences with a PerkinElmer spectrophotometer (Waltham, MA, USA) equipped with an attenuated total reflectance (ATR) accessory incorporating a diamond crystal as the internal reflection element. The spectra were collected in absorbance mode over the wavenumber range of 4000–500 cm^−1^ at a resolution of 0.9 cm^−1^ using samples of 0.005 ± 0.005 g. Each spectrum represented the mean of five successive scans. The interpretation of the FTIR spectra was based on a qualitative evaluation of changes in the positions, intensities, and bandwidths of signals associated with hydroxyl groups and polysaccharide structural features.

All FTIR measurements were performed using at least three independently prepared samples.

### 3.4. Analysis of the Morphological Structure of Starch–Phenolic Complexes

#### 3.4.1. Sample Preparation

Native, non-gelatinized starches were used exclusively for SEM analysis. Preliminary observations indicated that gelatinized samples formed compact, irregular masses that prevented meaningful visualization of starch granule morphology and digestion-induced surface changes. Therefore, native starches were selected to enable morphological evaluation at the granule level. This approach should be considered a limitation of the SEM analysis, as gelatinized starch would be more physiologically relevant.

##### Undigested Starch

Aqueous starch dispersions (5% *w*/*v*) were prepared using wheat, rice, potato, and maize starches (Sigma-Aldrich S5127; Sigma-Aldrich S7260; Chempur, Piekary Śląskie, Poland; Biomus, Lublin, Poland, respectively). The samples were centrifuged (MPW MED. INSTRUMENTS 351e, Warszawa, Poland) for 10 min at 3000× *g*. The resulting pellets were resuspended in 0.5 mL of distilled water, thoroughly mixed, and centrifuged again under the same conditions (10 min, 3000× *g*). The final residues were collected and freeze-dried (Alpha 1–4 LSCplus, Osterode am Harz, Germany) for further analysis.

##### Digested Starch

Aqueous starch dispersions (5% *w*/*v*) were prepared using wheat, rice, potato, and maize starches (Sigma-Aldrich S5127; Sigma-Aldrich S7260; Chempur, Piekary Śląskie, Poland; Biomus, Lublin, Poland, respectively). For preparing starch gels, 25 mg of starch was dispersed in 475 μL of distilled water. Native, non-gelatinized starches were used. The samples were subsequently subjected to in vitro enzymatic digestion using a mixture of pancreatic α-amylase and amyloglucosidase (PAA (40 KU/g)/AMG (17 KU/g)) prepared in 50 mM sodium maleate buffer (pH 6.0) containing 2 mM CaCl_2_. Digestion was carried out at 37 °C for up to 4 h in a water bath (MEMMERT WNE 14, Schwabach, Germany) under continuous stirring (170 rpm) to facilitate the hydrolysis of starch to D-glucose. After 240 min of digestion, 1 mL aliquots were withdrawn from the reaction mixture and immediately transferred to 20 mL of 50 mM acetic acid solution to terminate enzymatic activity. Following enzymatic incubation, the samples were centrifuged (MPW MED. INSTRUMENTS 351e, Warszawa, Poland) for 10 min at 3000× *g*. The resulting pellets were resuspended in 0.5 mL of distilled water, thoroughly mixed, and centrifuged again under the same conditions (10 min, 3000× *g*). The final residues were collected and freeze-dried (Alpha 1–4 LSCplus, Osterode am Harz, Germany) for further analysis.

Representative SEM images of the digested control were recorded at different magnifications to illustrate the general morphology of enzymatically digested starch. Owing to the extensive structural degradation of starch granules after digestion, separate control micrographs were not obtained for each botanical starch source. The images are therefore presented as representative qualitative controls.

##### Digested Starch–Polyphenol Complex

Aqueous starch dispersions (5% *w*/*v*) were prepared using wheat, rice, potato, and maize starches (Sigma-Aldrich S5127; Sigma-Aldrich S7260; Chempur, Piekary Śląskie, Poland; Biomus, Lublin, Poland, respectively). For preparing starch gels, 25 mg of starch was dispersed in 475 μL of distilled water. Epigallocatechin gallate (EGCG; Sigma-Aldrich, PHR1333, St. Louis, MO, USA) was added to the starch systems at concentrations of 10 mg for rice and potato starches and 20 mg for wheat and maize starches. The polyphenol and its doses were selected based on previous studies indicating the most favorable effects on starch digestibility and predicted glycemic index [12]. Native, non-gelatinized starches were used. The samples were subsequently subjected to in vitro enzymatic digestion using a mixture of pancreatic *α*-amylase and amyloglucosidase (PAA (40 KU/g)/AMG (17 KU/g)) prepared in 50 mM sodium maleate buffer (pH 6.0) containing 2 mM CaCl_2_. Digestion was carried out at 37 °C for up to 4 h in a water bath (MEMMERT WNE 14, Schwabach, Germany) under continuous stirring (170 rpm) to facilitate the hydrolysis of starch to *D*-glucose. After 240 min of digestion, 1 mL aliquots were withdrawn from the reaction mixture and immediately transferred to 20 mL of 50 mM acetic acid solution to terminate enzymatic activity. Following enzymatic incubation, the samples were centrifuged (MPW MED. INSTRUMENTS 351e, Warszawa, Poland) for 10 min at 3000× *g*. The resulting pellets were resuspended in 0.5 mL of distilled water, thoroughly mixed, and centrifuged again under the same conditions (10 min, 3000× *g*). The final residues were collected and freeze-dried (Alpha 1–4 LSCplus, Osterode am Harz, Germany) for further analysis.

#### 3.4.2. Surface Analysis by Scanning Electron Microscopy

Before imaging, the samples were coated with a thin layer of gold for 2 min using a sputter coater (108, Cressington Scientific Instruments Ltd., Watford, UK). Surface morphology was then examined with a scanning electron microscope (SEM; Hitachi 3400-N, Tokyo, Japan) equipped with a secondary electron (SE) detector. Micrographs were acquired at an accelerating voltage of 5 kV. SEM images of all samples were acquired at three different magnifications: 500×, 1000×, and 1500×.

SEM observations were performed using at least three independently prepared samples for each experimental system. Representative micrographs are presented in the manuscript.

Granule size measurements were performed using Fiji (Fiji Is Just ImageJ), an open-source image analysis software based on ImageJ (version 2.14.0/1.54f, National Institutes of Health, Bethesda, MD, USA). Before the analysis, images were calibrated using the scale bar. Representative dimensions of selected starch granules were obtained using Fiji software (version 2.14.0/1.54f) solely to illustrate the observed morphological features. No quantitative morphometric analysis or statistical evaluation of granule size (mean ± SD, *n*) was performed, and the reported dimensions were not intended for quantitative comparison among samples.

## 4. Conclusions

This study provides new insights into the structural characteristics of starch–polyphenol complexes formed from wheat, rice, potato, and maize starches under the experimental conditions used.

Apparent amylose content analysis revealed that incorporating different polyphenols significantly affected the apparent amylose content of wheat, rice, potato, and maize starches. In wheat starch, the control sample exhibited a significantly higher apparent amylose content than all starch–polyphenol complexes. For rice starch, a significant difference was observed between the epigallocatechin gallate complex and the remaining complexes. In potato starch, the greatest differences were found between the *p*-coumaric acid and naringenin complexes. In contrast, the epigallocatechin gallate complex in maize starch differed significantly from the *trans*-ferulic acid and quercetin complexes. These findings indicate that the effect of polyphenols on apparent amylose content is dependent on both the starch source and the type of polyphenol used. 

FTIR analysis suggested that polyphenols interact with starch primarily via noncovalent interactions, particularly hydrogen bonding, thereby altering starch molecular organization. The effects were more evident for epigallocatechin gallate and quercetin, while the extent of modification depended on the starch’s botanical origin. These findings suggest that the investigated starch–polyphenol complexes may have potential for the development of functional starch-based foods.

SEM analysis demonstrated that digestion in the presence of epigallocatechin gallate affected the morphology of starch granules in all investigated starches. The addition of this polyphenol appeared to prevent complete enzymatic degradation, as intact or partially preserved granules remained visible after digestion. In wheat starch, no typical surface erosion was observed. In contrast, rice and potato starches exhibited partially digested granules with evidence of degradation of some structural layers. Maize starch showed the most pronounced structural alterations, characterized by the formation of numerous pores on the granule surface and degradation of the internal layers. These observations suggest that epigallocatechin gallate modifies the susceptibility of starch granules to enzymatic hydrolysis and influences their digestion pattern.

It should be emphasized that, unlike the apparent amylose content and FTIR analyses, which included all investigated polyphenols, the SEM analysis was performed exclusively for native starch systems containing epigallocatechin gallate. Therefore, the observed morphological changes should be regarded as epigallocatechin gallate-specific and limited to native starch granules. These findings should not be generalized to the remaining polyphenols investigated or extrapolated to gelatinized or cooked starch systems, whose structural characteristics and enzymatic hydrolysis behavior differ substantially from those of native starch and therefore require further investigation using complementary analytical approaches.

Overall, the results demonstrate that incorporating the investigated polyphenols can modify selected chemical, molecular, and microstructural properties of the four botanical starches examined in the study. A better understanding of these starch–polyphenol interactions may facilitate the future development of functional starch-based products with improved nutritional and technological properties.

Further investigations involving gelatinized starch systems, a broader range of botanical starches and polyphenol concentrations, and complementary structural approaches, including X-ray diffraction and thermal analysis, would deepen understanding of the molecular architecture of starch–polyphenol complexes.

## Figures and Tables

**Table 1:** The apparent amylose content in wheat starch with and without the addition of different polyphenols.

SAMPLE CONTENT	POLYPHENOL	APPARENT AMYLOSE CONTENT [%]
Wheat starch without polyphenol	-	31.20 ^b^
Wheat starch with polyphenol	(*+*)-catechin	22.59 ^a^
	Epigallocatechin gallate	24.58 ^a^
	Naringenin	17.81 ^a^
	Quercetin	22.87 ^a^
	Kaempferol	22.28 ^a^

The results are presented as mean ± standard deviation (SD). Values marked with different letters differ significantly at *p* < 0.05.

**Table 2:** The apparent amylose content in rice starch with and without the addition of different polyphenols.

SAMPLE CONTENT	POLYPHENOL	APPARENT AMYLOSE CONTENT [%]
Rice starch without polyphenol	-	21.99 ^a,b^
Rice starch with polyphenol	Epigallocatechin gallate	26.95 ^b^
	Hesperidin	19.67 ^a^
	Naringenin	15.43 ^a^
	*Trans*-ferulic acid	20.26 ^a^
	*p*-coumaric acid	17.07 ^a^
	Quercetin	18.43 ^a^
	Kaempferol	18.35 ^a^

The results are presented as mean ± standard deviation (SD). Values marked with different letters differ significantly at *p* < 0.05.

**Table 3:** The apparent amylose content in potato starch with and without the addition of different polyphenols.

SAMPLE CONTENT	POLYPHENOL	APPARENT AMYLOSE CONTENT [%]
Potato starch without polyphenol	-	18.01 ^a,b^
Potato starch with polyphenol	(*+*)-catechin	22.28 ^a,b^
	Epigallocatechin gallate	20.16 ^a,b^
	Hesperidin	18.19 ^a,b^
	Naringenin	15.39 ^a^
	*p*-coumaric acid	22.66 ^b^
	Quercetin	20.92 ^a,b^
	Kaempferol	21.20 ^a,b^

The results are presented as mean ± standard deviation (SD). Values marked with different letters differ significantly at *p* < 0.05.

**Table 4:** The apparent amylose content in maize starch with and without the addition of different polyphenols.

SAMPLE CONTENT	POLYPHENOL	APPARENT AMYLOSE CONTENT [%]
Maize starch without polyphenol	-	24.83 ^a,b^
Maize starch with polyphenol	(*+*)-catechin	24.65 ^a,b^
	Epigallocatechin gallate	30.27 ^b^
	*Trans*-ferulic acid	19.10 ^a^
	Quercetin	22.19 ^a^
	Kaempferol	26.40 ^a,b^

The results are presented as mean ± standard deviation (SD). Values marked with different letters differ significantly at *p* < 0.05.

**Table 5:** Polyphenol concentrations and incorporation methods selected for individual starch systems based on previous digestibility and predicted glycemic index studies.

POLYPHENOL	STARCH			
	WHEAT	RICE	POTATO	MAIZE
(+)-catechin	20 mg, after gelatinization	–	10 mg, after gelatinization	5 mg, before gelatinization
Epigallocatechin gallate	20 mg, after gelatinization	10 mg, before gelatinization	10 mg, before gelatinization	20 mg, after gelatinization
Hesperidin	–	10 mg, before gelatinization	20 mg, after gelatinization	–
Naringenin	20 mg, after gelatinization	5 mg, before gelatinization	10 mg, after gelatinization	–
*trans*-ferulic acid	–	10 mg, before gelatinization	–	10 mg, after gelatinization
*p*-coumaric acid	–	10 mg, before gelatinization	10 mg, after gelatinization	–
Quercetin	5 mg, before gelatinization	10 mg, before gelatinization	20 mg, after gelatinization	5 mg, before gelatinization
Kaempferol	10 mg, before gelatinization	5 mg, before gelatinization	10 mg, after gelatinization	10 mg, after gelatinization

## Data Availability

The original contributions presented in this study are included in the article. Further inquiries can be directed to the corresponding authors.
